# “Sepsis brought him to his knees”: exploring the lived experiences and perspectives of sepsis survivors and family members to inform a sepsis public education campaign in Canada

**DOI:** 10.1186/s12889-025-22344-9

**Published:** 2025-03-31

**Authors:** Jeanna Parsons Leigh, Rebecca Brundin-Mather, Deirdre Walsh, Sara J. Mizen, Cynthia Sriskandarajah, Marie-Maxime Bergeron, Denise E. Werner, Kirsten M. Fiest

**Affiliations:** 1 Faculty of Health, School of Health Administration, Dalhousie University, Sir Charles Tupper Medical Building 5850 College Street, Second Floor, 2A01, Office 2A08, PO Box 15000, Halifax, NS B3H 4R2 Canada; 2 Department of Critical Care, University of Calgary, Calgary, Canada

**Keywords:** sepsis, Health campaigns, Health education, Health communication, Patient participation

## Abstract

**Background:**

Sepsis is a life-threatening complication of the body’s response to fighting an infection. The global burden of sepsis is incredibly high, accounting for an estimated 20% percent of all global deaths as well as high hospitalization costs and long-term multifaceted sequelae. As most sepsis starts in the community, public knowledge of sepsis is essential to rapid identification and medical intervention. The current study is part of multi-study collaborative research program. Following a scoping review and national survey to assess public knowledge of sepsis, we conducted focus groups to explore the lived experiences and perspectives of sepsis survivors and family members with the goal to inform development of a sepsis public education campaign.

**Methods:**

We co-designed a focus group guide covering three broad discussion topics: circumstances leading to sepsis, impacts of sepsis, and interactions with healthcare providers. Participants were purposively recruited through the previous national survey and through Sepsis Canada communications. We used a hybrid deductive-inductive approach to code transcripts and generate themes related to developing a sepsis public education campaign.

**Results:**

We conducted 11 focus groups with 32 participants. Participants’ median age was 53 years (Interquartile Range = 48, 64). Three-quarters (*n* = 23/32; 72%) self-identified as women, and all participants reported having some post-secondary education. All but one sepsis survivor were adults at the time of their diagnosis. We synthesized three overarching campaign messages from participant’s accounts of profound physical and mental impacts of sepsis and perceptions of health system failures: (1) sepsis is serious and common, (2) know the signs of sepsis, and (3) be health attentive and advocate health needs. Potential barriers to message uptake were: (1) sepsis is not well-known or easily understood, (2) perceptions that sepsis is not personally relevant, and (3) health messaging fatigue. Suggestions to effectively hook and draw public attention to sepsis centered on using personal stories and partnering with other health campaigns.

**Conclusions:**

Our analysis of participant’s lived experiences with sepsis suggest that public communications should aim to (1) improve sepsis symptom recognition, (2) foster perceptions that sepsis is personally relevant, and (3) cultivate and support health advocacy.

**Supplementary Information:**

The online version contains supplementary material available at 10.1186/s12889-025-22344-9.

## Background

Sepsis is a well-established threat to public health globally. Sepsis incidence and mortality estimates vary widely across and within countries, but in 2017 sepsis accounted for approximately 20% of all global deaths [1]. This means that worldwide 1 in 5 people are at risk of dying from sepsis, a statistic that only increases when sepsis progresses into more severe disease states [2]. Moreover, the hospital-wide cost of sepsis globally is estimated to be in the billions, although exact global figures are largely extrapolated from high-income countries making the data difficult to evaluate due to variations in healthcare systems [3–5]. The estimated total 1-year incremental cost of sepsis to the healthcare system in Canada’s largest province, Ontario, was estimated at 1 billion ($672.4 million for severe and $423.2 million for non-severe, sepsis) [6]. Survivors of sepsis often suffer a range of physical, cognitive, and psychological long-term health consequences [7–10]. Despite being a significant cause of hospital deaths and post-sepsis sequalae [11], sepsis remains largely unknown to the public worldwide [12]. The promotion of sepsis awareness has been a central call for public health action [13–15] and a core tenet of sepsis advocacy work. The World Health Assembly’s 2017 resolution to improve the prevention, diagnosis, and management of sepsis recommended that countries design “nationally relevant, specific messaging for educating the public and health care providers” about sepsis [16]. Canada’s response was to establish a single nationally coordinated research network—Sepsis Canada—to further understand the causes of sepsis, improve the prevention, detection, and management of sepsis, and support recovery and rehabilitation [17].

Although there are many large-scale efforts to raise the public profile of sepsis [18–20], there is limited published work describing the evidence supporting the design of sepsis public education campaigns. A lack of published formative research to substantiate the development of campaign goals, messages, formats and channels of communication, and intended audiences hinders comprehensive evaluation of their effectiveness [21]. Past research has shown that engaging interested parties, such as patients, in the research and policy-making process can enhance relevant and rigorous research [22], underscoring the need for a more collaborative approach in creating sepsis communications. The current study was conducted as part of Sepsis Canada’s evidence-based approach to creating a culture of sepsis awareness in Canada. The results of a previously published scoping review [12] and cross-national survey [23] highlighted poor public recognition of sepsis signs and symptoms, risk factors, and prevention measures. Against this backdrop, we conducted virtual focus groups with sepsis survivors and family members of sepsis patients (unrelated to recruited survivors) to help uncover factors that may be contributing to identified gaps in sepsis knowledge. By engaging individuals who had lived experiences with sepsis, we aimed to explore the key messages, channels, audiences, and barriers to consider when building a partnered sepsis public education campaign.

## Methods

### Study approach

We used a qualitative descriptive study design [24–26] to explore the lived experiences, perceptions, and perspectives of sepsis survivors and family members. Focus groups provided the opportunity to elicit shared and divergent viewpoints through discourse, potentially generating richer data than would otherwise be collected in one-on-one interviews [27].

Drawing on current literature [28] and previous experiences within our team conducting virtual focus groups during the Coronavirus Disease 2019 (COVID-19) pandemic [29], we established that focus group size should not exceed four participants and that focus groups with sepsis survivors should be conducted separately from focus groups with family members. The smaller group size and segmentation would help foster interpersonal comfort and relatability, and therefore responsiveness amongst participants. Two female team members (DW, patient partner; RBM, research staff) trained in qualitative methods conducted all focus groups using Zoom video conferencing platform (Zoom Communications, New York). DW facilitated the focus groups and RBM observed, took notes, and messaged DW through Zoom private chat to clarify participant comments and suggest additional probes. Participants could join the focus group via telephone or electronic device; those with video feed could have their cameras off or on. Immediately after each focus group, participants completed a demographic questionnaire via Qualtrics web-based survey platform (Qualtrics, Provo, UT). Participants were compensated with a $50 gift card. We stopped scheduling focus groups when the facilitator (DW), observer (RBM), and lead investigator (JPL) agreed that new insights specific to the study objectives were negligible.

A third-party company, Rev.com transcribed all focus group audio recordings. Two team members (RBM, SJM, research staff) reviewed, cleaned, and de-identified the transcripts before analysis. Fourteen participants (44%) chose to review their group transcript to confirm that all personally identifiable information was removed. We executed the study following the guidelines and regulations of the Research Ethics Boards at Dalhousie University (#2021 − 0950) and the University of Calgary (#21-5708) and reported study methods and results using the Consolidated Criteria for Reporting Qualitative Research [30] (Additional File 1). All participants electronically signed a consent form before focus group commencement.

### Participants

Most participants were recruited from a list of Canadian survey participants [23] who consented to be contacted about future research opportunities related to sepsis. The initial pool of eligible participants was English or French-speaking adults (≥ 18 years old) residing in Canada. We emailed a study invitation to those who reported that they had had sepsis (*n* = 33), or that a family member had had sepsis (*n* = 88). Family members were excluded if they had also experienced sepsis, but survivors were not excluded if a relative had experienced sepsis. Those recruited for the family member focus groups were unrelated to those recruited for the sepsis survivor focus groups, and vice versa (i.e., there were no intra-family dyads). To increase our sample of sepsis survivor participants, we posted study recruitment ads on Sepsis Canada’s Twitter feed and in their membership newsletter.

### Focus group guide design and testing

We developed a semi-structured focus group guide (see Additional File 2) comprising three overarching topics that would inform the development of a sepsis public education campaign: (1) circumstances leading to sepsis, (2) impacts of sepsis on participants’ life as a family member or survivor of sepsis, and (3) interactions with healthcare providers. The sepsis survivor interview guide was pilot tested with two team members (MMB, DEW, patient partners and sepsis survivors) to ensure suitability of core and probing questions. Minor changes were made to phrasing and sequence. The same guide was used with family members.

### Data management and analysis

We imported all transcripts into NVivo 12 (QSR International, Burlington, VT) for data analysis. Three team members (RBM, SJM, CS, research staff) independently familiarized themselves with the data by reading all transcripts and taking notes. The researchers then coded the first transcript individually. They compared their code lists, agreed on a consolidated codebook, and independently reanalyzed the first transcript before analyzing the second. They noted possible revisions to the consolidated codebook to incorporate new concepts and codes, which were refined by consensus. Analytic codes were identified both deductively from the focus group guide and inductively from the transcripts. The researchers then coded the complete dataset in duplicate. After all transcripts were coded, the researchers identified common patterns in the data [31] and with the lead investigator (JPL), collectively established the most salient themes pertinent to the study objectives. Resulting themes were organized to inform four key considerations in the development of a sepsis public education campaign: (1) focus, (2) barriers, (3) audience, and (4) formats and channels of communication. Exemplary quotes are provided in-text.

## Results

### Focus groups

**Fig. 1:**
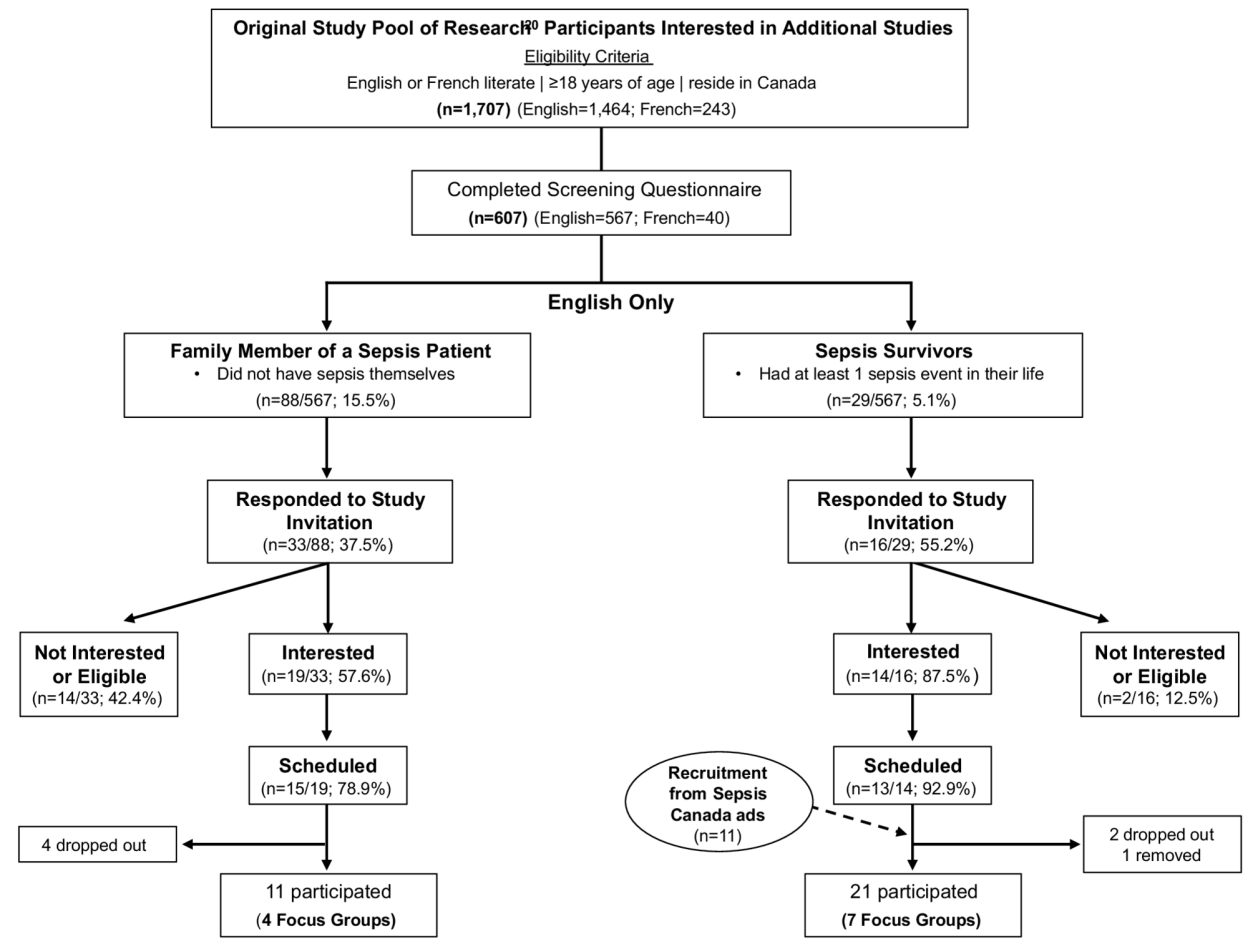
Participant recruitment flow

Figure 1 presents the flow of participant recruitment. We could not recruit enough French-speaking sepsis survivors or family members to conduct French language focus groups. Of the 117 study invitations we sent to English-speaking participants, 49 (41%) responded and 28 were scheduled. We scheduled an additional 11 sepsis survivors who contacted our team after seeing study recruitment ads placed in Sepsis Canada’s newsletter and Twitter feed. Six scheduled participants did not show or dropped out when the focus group started; one participant was removed due to geographic ineligibility. We conducted seven focus groups with sepsis survivors (21 participants) and four with family members (11 participants) between July 11 and September 12, 2022. The focus group size ranged from two to four participants. The average discussion time excluding breaks was 88 min (standard deviation ± 13 min). Nine (*n* = 9/32, 28%) participants did not use a device camera during the focus group; three of whom joined by telephone.

### Participant characteristics

Participants represented five of the six geographic regions in Canada [32]; just under half (*n* = 14/32, 44%) were from Ontario, the most populous province. Participants’ median age was 53 years (Interquartile Range (IQR) = 48, 64). Three-quarters (*n* = 23/32; 72%) self-identified as women, and all participants reported having some post-secondary education. All but one sepsis survivor were adults at the time of their diagnosis. Most (*n* = 30/32, 94%) were hospitalized for sepsis, and four (*n* = 4/32, 13%) reported having sepsis more than once. The most common suspected source of initial infection reported was gastrointestinal (9/30, 30%). Table 1 presents all participant characteristics.

**Table 1:** Focus group participant characteristics

		Total	Survivors	Family
Characteristics		*N* = 32(%)	*N* = 21(%)	*N* = 11(%)
**Region in Canada** ^**a**^				
	British Columbia	5(16)	4 (19)	1(9)
	Prairies	10(31)	6(29)	3(27)
	Ontario	14(44)	10(48)	5(45)
	Québec	1(3)	1(5)	0
	Atlantic	2(6)	0	2(18)
	Territories	0	-	-
**Age category**,** years**				
	Median, (IQR)	53 (48, 64)	54 (49, 67)	51 (45, 56)
	18–29	1(3)	1(5)	0
	30–44	5(16)	2(10	3(27)
	45–64	19(59)	12(57)	7(64)
	≥ 65	7(22)	6(29)	1(9)
**Gender**				
	Women	23(72)	15(71)	8(73)
	Men	8(25)	5(24)	3(27)
	Two-spirited	1(3)	1(5)	-
**Ethnicity** ^b^				
	Asian	1(3)	0	1(9)
	Black	1(3)	1(5)	0
	Indigenous	2(6)	2(10)	0
	White	29(91)	19(90)	10(91)
**Education** ^c^				
	Highschool	0	-	-
	Trade/some post-secondary	7(22)	5(24)	2(18)
	Post-secondary degree	24(75)	15(71)	9(82)
**Sepsis Occurrences**				
	Once	28(88)	18(86)	10(91)
	Multiple	4(13)	3(14)	1(9)
**Time since sepsis occurrence** ^d^				
	≤ 5 years or less	14(44)	10(48)	4(36)
	6–10 years	5(16)	3(14)	2(18)
	> 10 years	10(31)	7(33)	3(27)
**Setting first visited with symptoms** ^e^				
	Family doctor	2(7)	1(5)	1(11)
	Walk-in Clinic	6(24)	5(24)	1(11)
	Emergency department in hospital	17(57)	12(57)	5(56)
	Already in hospital	5(17)	3(14)	2(22)
**Hospitalized**				
	Yes	30(94)	19(90)	11(100)
	No	2(6)	2(10)	-
**Suspected initial insult of infection** ^f^				
	Gastrointestinal	9(32)	5(25)	4(50)
	Oral	1(4)	0	1(13)
	Respiratory	1(1)	0	1(13)
	Skin	6(6)	6(30)	0
	Surgery/HAI	7(25)	6(30)	1(13)
	Urinary	4(14)	3(15)	1(13)
**Suspected sepsis-associated death**	Yes	-	-	3 (27)

^a^ Based on Statistics Canada’s standard geographic classification (2016) (Statistics Canada. (May 22, 2018). Table C List of geographical regions of Canada with codes, 2016. Statistics Canada. Retrieved July 1, 2024, from https://www.statcan.gc.ca/en/subjects/standard/sgc/2016/introduction)

^b^ Values exceed the number of participants as participants could select multiple options for ethnicity.

^c^*N* = 30; missing data from 1 sepsis survivor.

^d^ Participants who had more than one occurrence of sepsis incident reported the month and year of their last occurrence. *N* = 28; missing data from 1 sepsis survivor and from 2 family members.

^e^*N* = 29; missing data from 2 family members.

^f^ Categories were derived from the following question in the participant demographic questionnaire, *what type of infection led to the complication of sepsis (if known)*? *N* = 28; missing data from 1 survivor and 3 family members

### Campaign focus

Across focus groups, three overarching messages or calls to action were highlighted as important in a sepsis public education campaign: (1) sepsis is serious and common, (2) know the signs and symptoms of sepsis, and (3) be attentive to your health and advocate your needs.

#### Message #1: sepsis is serious and common

All focus groups stressed that the public needs to better understand that sepsis has severe medical consequences, impacting both short- and long-term health. Depicted in Table 2, the breadth and depth of impacts described by our participant sample were profound. Frequently noted impacts on physical health included fatigue and weakness, pain, memory loss, and other impairments like kidney failure, sensory loss, and amputation or organ removal.

I used to have a lot of energy. Now, I feel like an old cell phone. It doesn’t charge properly. It doesn’t keep a charge. All of a sudden, it’s done. I would say I get about four functional hours a day. (FG04-SS, Par 10)

**Table 2:** Participant reported impacts of sepsis on the sepsis sufferer^a^

Health Domain	Impact Description
**Physical**	
	• Chronic pain • Chronic medication needs • Fatigue, exhaustion, and/or muscle weakness • Organ or limb loss / damage / dysfunction • Sleep disturbances and trouble sleeping • Weakened immune system
**Mental**	
	• Acute trauma and post-traumatic stress disorder • Anger and blame towards healthcare providers • Anxiety (prolonged) • Depression • Fear of getting sick again • Feeling like a burden on loved ones and on health care providers • Guilt and self-blame for getting sepsis • Stress (prolonged) • Unable to perform previous hobbies or activities
**Social**	
	• Aesthetic changes to the body such as skin necrosis • More empathy for others with chronic illness • Strained relationships with friends and family
**Financial**	
	• Employment changes (job loss, career shifts, early retirement) • Out-of-pocket healthcare expenses for rehabilitation

^a^ Impacts are multifaceted and can affect the health and well-being of sepsis sufferers in domains other than the one the impact is classified in. For example, chronic pain while classified as a physical impact can also impact mental, social, and financial wellbeing.

Six (*n* = 6/21; 28%) sepsis survivors with a median age of 53 years experienced job loss or work adjustments in the months following their sepsis diagnosis. Psychological outcomes were prominent, including stress, depression, and anxiety (see Table 2); a quarter of participants (*n* = 8/32; 25%) mentioned struggles with post-traumatic stress disorder. Survivors talked about the difficulty managing persistent symptoms, which, as one participant commented, was compounded by perceived disbelief from others who “think you’re making (it) up” (FG06-SS, Par 16).

Groups considered optimal approaches to communicate the seriousness of sepsis emphasizing that sepsis is not rare, and everyone is at risk. Described by one participant, sepsis is “the quiet killer that’s infiltrating our society” (FG05-SS, Par 14). Reporting risk and prevalence statistics was proposed as a potentially impactful strategy.

If you were to say 3% of all the people who go into the hospital with sepsis may result in death or something like that, even though it’s only 3%, that’s still a shocking number. And considering… you’re in the hospital where you’re receiving care and it could still result in death, that would tell me that it’s far more serious than what I would think. (FG10-FM, Par 27)

However, discussions about conveying the seriousness of sepsis elicited differing perspectives on using ‘scare tactics’ (e.g., risk of death) and the effectiveness of this approach. As one participant stated:

You don’t want to go the fearmongering route that if you’re septic, you could die. It’s no (pause), if you get sepsis, it is treatable, but there are long term ramifications, so it’s best to get it early. So, managing that, you don’t want to scare people. (FG07-SS, Par 19)

Yet others felt that scaring people might be unavoidable to appropriately underscore the seriousness of sepsis. One participant even suggested that fear could be an important motivator for getting symptoms checked.

#### Message #2. know the signs and symptoms of sepsis

In recounting their own experiences with sepsis, participants described a range of precipitating symptoms such as fever, general malaise, hot to the touch, delirious, unable to urinate, mental confusion, racing heart, swelling, shivering, shaking, and pain—the gravity of which were not always immediately recognized. In all focus groups, participants discussed that signs were either discounted, misunderstood, or not disclosed, which might have influenced the course of their illness and experiences in the healthcare system. Most significant were delays in diagnosis or treatment initiation after entering the healthcare system, as well as cases of nosocomial infection (*n* = 5/30; 17%). Six of the 17 (35%) participants in our sample who went to the Emergency Department (ED) with symptoms were evaluated and told to go home. One family member visited the ED at three hospitals before her teenaged son was admitted.

Participant’s descriptions suggested that the late detection and treatment of sepsis had resounding mental and emotional impacts and for some, was particularly damaging to their trust and confidence in healthcare professionals. Expressions of guilt and blame resonated across focus groups, alluding to the complexities in assessing when medical attention is needed. While some sepsis survivors conveyed their reluctance “burdening” their family or healthcare providers, family members voiced their concern and frustration at the initial lack of symptom disclosure or missed signs. One family member captured these sentiments when describing her family not noticing an infection on her father’s back.

We kicked ourselves afterwards because we thought…(pause). We were checking his feet, we were worried about his gums, but we never thought to ask him about that (his back) because why would you, right?” (FG11-FM, Par 31).

Blame was also extended to healthcare providers as many survivors and family members described feeling dismissed when seeking treatment for initial symptoms. Frustration only mounted once sepsis was identified as they described feeling blamed for not seeking care sooner, even if the infection was acquired in the hospital.

It’s an infection and they try to twist it so that, “You have to take care of yourself better.” And I says (sic), “Wait a minute. You did the surgery and I ended up with an infection. Why weren’t your instruments clean? Were your uniforms clean? Did you clean the operating room? I didn’t have the infection before you did the surgery. (FG01-SS, Par 2)

#### Message #3: be attentive to your health and advocate your needs

Most survivors and family members in our sample reported greater vigilance and monitoring of their own health and that of family members because of their sepsis diagnosis and experiences in the healthcare system. While it positively propelled some family and friends to be more proactive with preventive health measures, most participants expressed varying degrees of concern, anxiety, or fear of getting sick.

Once you’ve had it (sepsis) once you don’t want to go through it again, so like everyone else (in the focus group) says, (it) might be hypochondriacal, but you’re always watching for, “Okay, is this getting worse? Is it getting better? Do I need to get it checked out? Or can I still take care of it myself?” (FG03-SS, Par 06).

Personal health awareness was discussed as essential to recognizing indicators of severe illness and knowing when to seek medical attention. One focus group (FG08-FM) brainstormed sepsis public education campaign slogans such as “Tune into Yourself” (Par 23) and “Check in, don’t check out” (Par 24). All groups iterated the importance of telling someone if feeling unwell, and that symptoms get worse the longer they are ignored. This was felt to be exceedingly important for infection that can escalate in severity over a very short period.

I think that knowing some symptoms is helpful, but even more importantly, for people to take symptoms seriously, and not try to sweat things out, or minimize things because if it gets worse, it’s going to get worse quickly to where you’re not able to function. (FG03-SS, Par 08).

Self-advocacy was a key theme, particularly in the context of interactions with health providers. While this was tied to vigilance and bodily awareness, many participants in our sample expressed being failed by the healthcare system at one or more points throughout their sepsis journey. Feeling alone, misheard, and having unmet care or information needs created additional struggles beyond their sepsis diagnosis.

I felt really alone, not alone, like sad, but alone in managing it. I felt like I had to be a strong advocate for myself. I don’t really recall anybody kind of walking beside me or supporting me in that, in the healthcare role at all. (FG04-SS, Par 11)

A few participants were deliberate in learning how to advocate to ensure they could obtain and understand health information to make decisions that were appropriate for themselves or their family member. Much of this learning came from self-directed internet searches, as many participants described a lack of information from healthcare providers. There was a shared sense of exhaustion in describing how discouraging it was to constantly push healthcare professionals to get treatment or information, particularly when already unwell.

### Campaign barriers

Our analysis identified three inter-related themes that participants believed could hinder the uptake of sepsis public education communications: (1) sepsis is not well-known or easily understood, (2) sepsis does not affect me, and (3) health message fatigue.

#### Sepsis is not well-known or easily understood

Participants acknowledged that signs of sepsis resemble symptoms of common illnesses and therefore are often difficult to identify as sepsis.

We had heard that (my mother-in-law) wasn’t really feeling well, so we thought maybe she had a bit of a cold or flu, or maybe just kind of run down and tired, that kind of thing. And then about two days later, she was in the hospital (with sepsis). (FG08-FM, Par 24)

Without some level of familiarity, many participants felt that any exposure to messages about sepsis may be received indifferently. Over half (*n* = 19/32, 59%) of the participants in our sample indicated that they had heard of sepsis prior to their experience but very few reported having much knowledge about sepsis. Most learning occurred after diagnosis, and rarely from their healthcare team. When asked to recall how sepsis was described to them in hospital, participants commonly reported being told that they, or their family member, had had a blood infection. Although a few participants noted that they received a good explanation of sepsis from a healthcare provider, most sought information from other sources, predominantly the Internet. Wanting to have clearer explanations of sepsis as well as of post-sepsis effects were central themes in discourse around information gaps.

#### Sepsis does not affect me

A second identified barrier was the suggestion that people tend to disregard messages that they perceive as irrelevant to their life. One family member illustrated this point in describing that her attention to COVID-19 social media posts was only heightened when she came down with COVID-19.

Once that event had occurred in my life, I actually stopped scrolling past it and read the statistics, because I was one of them. So, I think that relatability to the content is a really important part of it. (FG08-FM, Par 22)

This participant went on to acknowledge that the major challenge is figuring out how to disseminate information in ways that will catch the attention of people who cannot relate to sepsis because they have not been directly impacted. As described by one survivor, the response “it’ll never happen to me” (FG06-SS, Par 16) is common. Similarly, another participant speculated that fear could also contribute to message avoidance because “people don’t want to hear about problems that aren’t already there, right?” (FG08-FM, Par 24).

#### Health message fatigue

All focus groups acknowledged the challenge in raising awareness about sepsis in the wake of the COVID-19 pandemic when the public was inundated with information about personal health and disease prevention. Participants debated whether the constant exposure to health messages might dampen rather than stimulate attention. This was most prominent in discussing sepsis prevention. Although all focus groups could see the general logic in messaging sepsis as a preventable illness (through infection prevention), the notion also engendered some confusion. Questions like “Can sepsis be prevented”, “How would vaccination help”, and “Is there a vaccine for sepsis” were striking signals that sepsis prevention may require clearer messaging to connect infection and sepsis. Moreover, although sometimes hotly debated, most participants felt that because of COVID-19, “people are sick and tired of hearing about hand washing and (infectious) disease measures as prevention for anything” (FG06-SS, Par 18). Still, there were participants who saw COVID-19 as an opportunity for sepsis communications, particularly in the context of long COVID which seems to mirror the recovery trajectory of many sepsis survivors.

I think that’s where we have some room to talk about the sepsis and long COVID links. Because I think COVID is a buzzword that people are more attuned to. (FG06-SS, Par 18)

### Campaign audience

Participants unanimously stated that everybody needs to be aware of sepsis, often citing anecdotes that showed the diversity of people affected by sepsis.

My friend knew that 19-year-old athlete who went to the (ED). They told her she was fine. She went home and died pretty soon afterwards. I think it’s anybody because any infection can start this (sepsis) off. (FG 06-SS, Par 17)

However, in talking about who a campaign should reach, most groups identified specific sub-populations to target, typically individuals thought to be at higher risk of developing sepsis, including individuals with immunosuppression, older adults, substance users, and pregnant women. A few participants suggested using sepsis incidence data to identify priority groups. Unprompted, many participants–especially those who experienced delayed diagnoses and/or poor communications with their healthcare team–emphasized the need to educate healthcare providers about sepsis, including post-sepsis syndrome. They noted that education should target hospital-based as well as community-based healthcare providers, including family physicians and personal support and homecare workers.

I worked as a personal support worker and there wasn’t the education. I noticed in the hospitals, there wasn’t even the understanding with occupational therapy and physiotherapy. So, they need to grasp an understanding of the ramifications (of sepsis). (FG07-SS, Par 20)

Children were a sub-population that all focus groups mentioned targeting. Participants commented that it was important to start education early to learn signs and symptoms and the appropriate action to take. Children were seen as sponges for information and could raise concern about unusual behavior or symptoms in family members.

### Campaign formats and channels of communication

Participants agreed that a range of formats and channels was needed to effectively target different segments of the population and disseminate messages as broadly as possible. We categorized participant suggestions into four broad communication channels:

- community-based (buses, health clinics, hospitals, billboards, and pharmacies);
- traditional media (magazines, newspapers, radio, and television (mainstream and online));
- schools (healthcare programs, first-aid training, and youth/children curriculum); and
- digital media (websites, social media like Twitter, Facebook, and Tik Tok).

Although some novel ideas were brainstormed, such as contacting 3M Canada to add a sepsis caution label to bandage boxes, social media was the most frequently mentioned approach due to its low cost, ease of development, and broad reach, particularly among younger adults, who were perceived to be least knowledgeable about sepsis. Integrating interactive posts like a knowledge quiz—what is true about sepsis—could foster learning in quick and entertaining ways.

Across focus groups, participants highlighted pharmacies as an ideal (non-digital) location for sepsis education, citing their accessibility, the presence of knowledgeable healthcare professionals, and the fact that most individuals frequent a pharmacy at some point. Posters and brochures were typically mentioned, and one participant suggested including information about sepsis with antibiotic prescriptions. Another participant also remarked that a sepsis awareness day should be established, acutely illustrating the challenge of effectively reaching a broad audience as annual campaigns promoting World Sepsis Day have been around for over a decade.

Most focus groups indicated that a sepsis public education campaign needed to create a ‘hook’ to overcome public apathy and draw people’s attention. One frequently noted strategy was to elicit empathy through personal stories which were believed to resonate most profoundly with people and therefore more likely to be heard. Participants described opportunities they had to start conversations by sharing their stories with wider audiences, including posting on websites, speaking to neighbors, and presenting to medical and nursing students.

And (sharing stories) makes a huge, huge difference. I’ve received letters from people saying that the stories that I shared saved their mother or saved their niece because they were actually reading my post while they were talking to their sister on the phone about the child, and the signs were there. (FG05-SS, Par 15)

At the same time, as one sepsis survivor reminded their group, personal narratives would be most impactful if their relevance to the audience’s circumstances could be shown. This related to a second strategy to ‘hook’ people, which was to compare sepsis to other more well-known conditions like heart disease, cancer, and stroke, perhaps through comparing risk and incidence statistics. This complemented another suggestion to mirror the strategies used by other public education campaigns perceived as successful; the FAST campaign for recognizing the signs of stroke [33] was highlighted by most groups as a campaign that reached many people because people remember the acronym. Partnering with other causes was also noted as a way to capture attention as sepsis is a complication of many other conditions.

Our cause would be secondary to what their cause is. And it (sepsis) could be a complicating factor of all of their ailments as well. So maybe we could be looking at some strategic partnerships of cancer and all the other avenues. (FG05-SS, Par 14)

## Discussion

After conducting a scoping review of global literature [12] and a national cross-sectional survey [23] to assess public knowledge of sepsis, we conducted a focus group study to explore lived experiences and perspectives of sepsis survivors and family members to inform the development of a sepsis public education campaign. From 11 focus groups with 32 participants, we identified possible topics, audiences, and formats and channels for communicating about sepsis. Content recommendations centered on (1) knowing that sepsis is serious and common, (2) knowing the signs and symptoms of sepsis, and (3) encouraging health attentiveness and self-advocacy. We also identified several barriers that could hinder the uptake of messaging, which require serious consideration in designing, disseminating, and evaluating education interventions.

### Barriers to early recognition of sepsis

Sepsis is typically difficult to identify as symptoms are often nonspecific and can easily be misattributed to a less serious illness, particularly in initial stages [9, 34, 35]. Most participants in our study had heard of sepsis, but few reported knowing signs of sepsis, prior to their, or their family member’s diagnosis. They described a range of initial symptoms which typically worsened before they sought help; the challenge for many was labelling their symptoms and knowing when to seek medical attention. Andersen’s General Model of Total Patient Delay [36–38] widely applied to cancer diagnosis may be useful in understanding our findings. Andersen’s model proposes a series of sequential stages—appraisal, help-seeking, diagnosis, treatment— from symptom onset to the start of treatment. Varied sociodemographic factors, health experiences, and health beliefs can mediate appraisal and decision processes at each stage. Delays during the prehospital period have been previously examined for sepsis [39, 40] as well as for other time-sensitive conditions like stroke [41, 42] and heart attack [43, 44], which tend to have more unique clinical indicators when compared to sepsis [35]. Common reasons for delaying seeking help included experiencing symptoms inconsistent with promoted disease symptoms, attributing symptoms to other problems, and underappreciating the significance of symptoms. Familiarity with the symptom profile and perceiving symptoms as severe were both factors associated with shorter time intervals, highlighting the need for greater sepsis knowledge.

Some participants in our study objected to using the word “delay” to characterize the time before seeking help, as it was perceived as blaming the patient. This sense of blame was particularly evident in participants who experienced significant medical contact delays in the ED, either by being sent home or having to wait for extended periods. Delays in sepsis detection and treatment are not unique to our study. Filbin et al. found that a third of septic shock patients presented to the ED with “vague symptoms”, which impacted the time-to-antibiotics compared to those with explicit symptoms of infection [45]. ED delays can result from challenges (1) triaging acuity without a cardinal marker of sepsis, (2) detecting subtle drops in vital signs, particularly when the ED is busy or overcrowded, and (3) assessing patients with comorbidities and multiple or unclear complaints [46]. These challenges are further compounded by high patient caseloads, staff shortages, lack of rapid diagnostic tests, insufficient training, and lack of familiarity with sepsis guidelines [47]. An analysis of a decade of Canadian sepsis-related medico-legal cases further corroborates deficiencies in assessing patients for sepsis [48]. Unfortunately, delays in diagnosis and appropriate management of sepsis reduces the window for optimal care [16] and impacts patient and family trust and confidence in the health system. Understanding the scope of patient and health system factors that could contribute to treatment delay from symptom onset is critical to improving early recognition and management.

### Target audiences for sepsis education

When considering priority audiences for sepsis education, perceived failures in sepsis diagnosis and treatment likely influenced many participants in our study to suggest healthcare providers. Participants saw a critical need for instruction targeted at both hospital- and community-based providers, covering the continuum of sepsis care—from diagnosis and hospital discharge to post-hospital rehabilitation, which has been shown in the literature to be often overlooked in discharge planning [49]. Health professionals are widely recognized as trusted sources of health information [50, 51], and quality clinician-patient communication linked to better patient satisfaction and health outcomes [51]. The WHO resolution calls for healthcare providers to increase awareness of sepsis by using the term “sepsis” in their communications with patients and families [5]. Yet, in our study, participants described few instances in which members of their care team used the term ‘sepsis’ or explained what sepsis was, often leading to confusion and incomplete knowledge about sepsis and post-sepsis expectations. Past research has shown this to be a common experience for patients, as clinicians assume that the illness would not be understood outside of the medical field [46]. Despite the challenges in detecting sepsis early, a strategic approach to improving sepsis literacy and patient outcomes may still involve targeting healthcare providers to enhance their knowledge and communication about sepsis.

In addition to healthcare providers, our study participants noted other potential audience segments to target, largely based on perceptions of populations at increased risk of sepsis such as parents of infants, older adults, and those who have underlying medical problems [1, 52, 53]. However, children were mentioned in all focus groups for sepsis education with the dual purpose to empower children to become health literate and to increase their capacity to recognize warning signs of sepsis. Globally, disease prevention has been effectively introduced into a school-based curriculum of children for other conditions such as HIV/AIDs [54], cardiovascular disease [55], stroke [56], and infections like COVID-19 [57]. Existing campaigns to raise awareness of sepsis in schools—such as the American “End Sepsis” education program [58] and the Scottish SEPS_IS Engagement Project [59]—merit closer review and evaluation. Sub-populations in Canada identified as least knowledgeable about sepsis in a recent national survey [23] were young adults (between 18 and 29) and males. Therefore, interventions directed towards younger populations may be most effective in raising awareness.

### Health message fatigue

A key barrier to the uptake of a sepsis public education campaign identified in our study was health message fatigue. Prior to the COVID-19 pandemic, research widely documented public fatigue toward prominent health promotion and disease prevention campaigns (e.g., tobacco use, obesity) [60, 61]. However, the proliferation of health messaging during the COVID-19 pandemic significantly advanced research on message fatigue. Message fatigue refers to the feeling of being overwhelmed by persistent exposure to similar or redundant messages, which can become counterproductive by fostering resistance to the original message intent [60]. Moreover, message fatigue generally increases, and motivation to act decreases, when the message content is dense and overly elaborative [62]. Several messages in current sepsis campaigns, particularly those around prevention, mirror those disseminated during COVID-19, including reminders of hand washing and vaccination. Additionally, as COVID-19 is a viral infection that can lead to sepsis, there is an increasing amount of information about COVID-19 on public-focused sepsis websites. Close attention should be paid to the types of information that may cause message fatigue, particularly as they related to COVID-19 [63]. Furthermore, as message fatigue is related to individual factors such as health status and health literacy, using interactive tools such as AI chatbots—capable of accommodating individual motivations and abilities to tailor messages and increase personal relevance [62]—as well as leveraging healthcare providers like pharmacists to educate patients, should be explored.

### Format and channels of communications

World Sepsis Awareness Day (September 13th) has been a key milestone in global sepsis awareness and advocacy efforts. However, in general, many health awareness days exist without much evaluation [21, 64]. To make these efforts more effective, it is essential to conduct formative research with target audiences and test messages to ensure they are relevant, understandable, and engaging. If implemented as best practice, barriers like message fatigue can be prevented and the way information is shared can be improved [65, 66]. Similarly, evaluating the reach and impact of public communications is essential to assessing the success of message delivery, impact, and overall campaign objectives. Given the limited evaluation of sepsis campaigns in the literature, exploring potential evaluation methods with individuals with lived experience of sepsis, along with approaches used in other campaigns, such as those for stroke [67–70], lung cancer [71], heart health [72, 73], and COVID-19 [74], may be beneficial. This is particularly important in determining whether increased awareness and knowledge translates into appropriate behavior. For example, Morrow et al. found that despite experiencing or witnessing stroke symptoms, most participants in their study were unable to apply their knowledge of the FAST campaign [75] to reduce time to hospital.

Our findings indicated that messages should be innovative, personalized, and strategically shared. Many sepsis survivors and family members advocated for eye-catching text and graphic promotional content distributed through various channels. A recent sepsis-specific study found that both text-based and graphic formats effectively fostered informed decision-making as well as risk and health literacy; however, the text-based format was associated with higher levels of understanding, but only among participants under 60 years of age [76]. Key messages to promote included highlighting the danger of sepsis in relation to infection and other diseases, avoiding the colloquial term “blood poisoning”, and conveying the rapidity and severity of sepsis. To add to this list, our findings underscored the use of statistics to capture attention.

### Limitations

A key strength of this study was the engagement of patient partners in co-designing the focus group guide and in facilitating the focus groups which helped elicit rich descriptions of common and unique experiences as well as innovative strategies to raise sepsis awareness. However, there are several limitations to consider when interpreting our study findings. First, we purposively recruited from a convenience sample which did not result in demographic diversity. Participants were predominantly self-reported White women over the age of 45. Recruiting more individuals who were younger, men, or from indigenous or racial or ethnic minority groups may have resulted in different discourse around healthcare experiences and perspectives on raising sepsis awareness. Second, for some participants, the length of time since their sepsis event may have affected their recollections. Third, we did not confirm participants’ self-reported sepsis diagnosis by extracting diagnostic codes from health administrative data. Additionally, sepsis survivors recruited through Sepsis Canada media ads may be more engaged in advocacy work, possibly introducing bias. Finally, we did not conduct focus groups with healthcare providers. Asking comparable questions to healthcare providers could provide a more comprehensive understanding of sepsis diagnosis, care, and the information gaps identified by our participants, particularly those related to the healthcare system.

## Conclusions

Despite its threat to global health, sepsis remains largely unknown to the public. Through virtual focus groups with sepsis survivors and family members, we explored factors contributing to public knowledge gaps and identified effective ways to address them. By pinpointing key target audiences, accessible formats, and digestible messages, this research highlighted opportunities to innovatively enhance sepsis communications. Overcoming barriers to information uptake, such as health messaging fatigue, will be critical to developing an educational campaign that empowers the public to recognize the signs and urgency of sepsis.

## Electronic Supplementary Material

Below is the link to the electronic supplementary material.

Supplementary Material 1

Supplementary Material 2

Supplementary Material 3

## Data Availability

The dataset for the current study is not publicly available as we did not secure permission from the focus group participants to share the de-identified dataset publicly. Requests for the de-identified data can be directed to the institutional research ethics boards overseeing the conduct of the study via the principal investigator, Dr. Jeanna Parsons Leigh (jparsonsleigh@dal.ca).

## Abbreviations

COVID-19: Coronavirus Disease 2019
ED: Emergency Department
FG: Focus Group
FM: Family Member
HAI: Hospital-acquired infection
IQR: Interquartile range
HIV/AIDs: Human immunodeficiency virus/acquired immunodeficiency syndrome
Par: Participant
SS: Sepsis Survivor
WHO: World Health Organization
