# Supplementary material for: “Sepsis brought him to his knees”: exploring the lived experiences and perspectives of sepsis survivors and family members to inform a sepsis public education campaign in Canada
Source: BMC Public Health. 2025 Mar 31;25:1211. doi: 10.1186/s12889-025-22344-9 (PMC11956199; doi:10.1186/s12889-025-22344-9)
Supplement: Supplementary file 1 — Supplementary Material 1 [file 12889_2025_22344_MOESM1_ESM.pdf]

Additional Files List

Additional File 1. Consolidated criteria for Reporting Qualitative research (COREQ) Checklist

Additional file 2. Semi-Structured Focus Group Guide.
