# Supplementary material for: “Sepsis brought him to his knees”: exploring the lived experiences and perspectives of sepsis survivors and family members to inform a sepsis public education campaign in Canada
Source: BMC Public Health. 2025 Mar 31;25:1211. doi: 10.1186/s12889-025-22344-9 (PMC11956199; doi:10.1186/s12889-025-22344-9)
Supplement: Supplementary file 2 — Supplementary Material 2 [file 12889_2025_22344_MOESM2_ESM.pdf]

Additional File 1
Consolidated criteria for Reporting Qualitative research (COREQ) Checklist[30]

Guide question	Responses	Location in Manuscript
**Domain 1: Research team and reflexivity**		
Personal Characteristics		
Which author/s conducted the interview or focus group?	Rebecca Brundin-Mather, MASc<br>Deirdre Walsh, BSc	Methods
What were the researcher's credentials? E.g. PhD, MD	Jeanna Parsons Leigh (JPL), PhD<br>Rebecca Brundin-Mather (RBM), MASc<br>Deirdre Walsh (DW), BSc<br>Sara J. Mizen (SJM), MA<br>Cynthia Sriskandarajah (CS), MPH<br>Marie Maxime Bergeron (MMB), RD<br>Denise E. Werner (DEW), RN<br>Kirsten M. Fiest (KMF), PhD	Title Page
What was their occupation at the time of the study?	Jeanna Parsons Leigh, Assistant Professor<br>Rebecca Brundin-Mather, Research Associate<br>Deirdre Walsh, Patient Partner<br>Cynthia Sriskandarajah, Research Assistant<br>Sara J. Mizen, Research Assistant<br>Marie Maxime Bergeron, Patient Partner<br>Denise E. Werner, Patient Partner<br>Kirsten M. Fiest, Associate Professor	Not reported in manuscript
Was the researcher male or female?	Female: 100%<br>Male: 0%	Methods
What experience or training did the researcher have?	DW completed the PaCER program at the University of Calgary and has lived experience with chronic illness; MMB and DEW have lived experience with sepsis and work in healthcare; all other authors have training in qualitative methods through graduate studies and employment.	Methods
Relationship with participants		
Was a relationship established prior to study commencement?	No. Most participants were recruited from a previously conducted anonymous survey where participants could indicate interest in being contacted about future studies related to sepsis.	Methods

What did the participants know about the researcher? e.g. personal goals, reasons for doing the research	Eligible participants were sent an invitation for the study which outlined why they were being invited, the study objectives, the study design, and the study's primary investigators (JPL, KM). They could email questions about the study to the study coordinator (RBM). Full details of the study and study team were provided in the informed consent form.	Not reported in manuscript
What characteristics were reported about the interviewer/facilitator? e.g., Bias, assumptions, reasons and interests in the research topic	Participants were made aware that the facilitator was a patient partner with Sepsis Canada, and the observer was a research associate on the study.	Not reported in the manuscript
**Domain 2: Study design**		
Theoretical framework		
What methodological orientation was stated to underpin the study? e.g., grounded theory, discourse analysis, ethnography, phenomenology, content analysis	Qualitative Description study design (see references 24-26)	Methods
Participant Selection		
How were participants selected? e.g., purposive, convenience, consecutive, snowball	We purposively selected participants from a convenience sample of participants from a previous study	Methods
How were participants approached? e.g., face-to-face, telephone, mail, email	We approached potential participants via e-mail; some participants contacted us directly after seeing recruitment ads for the study through Sepsis Canada.	Methods
How many participants were in the study?	32	Results
How many people refused to participate or dropped out? Reasons?	6 scheduled participants dropped out due to family emergency, ineligibility, or reasons unknown.	Methods<br>Figure 1
Setting		
Where was the data collected? e.g., home, clinic, workplace	Focus Groups were conducted virtually via institutional Zoom license	Methods
Was anyone else present besides the participants and researchers?	No	Not reported in manuscript
What are the important characteristics of the sample? e.g., demographic data, date	Demographic and health outcome data	Results<br>Table 1

Data collection		
Were questions, prompts, guides provided by the authors? Was it pilot tested?	The focus group guide was piloted tested with 2 patient partners (MB, DEW). The focus group guide questions were not provided a priori to participants, except for 2 individuals so they could assess their comfort level with the questions and ascertain they could contribute to the aims of the focus group.	Methods Multimedia Appendix 2
Were repeat interviews carried out? If yes, how many?	No	Not reported in manuscript
Did the research use audio or visual recording to collect the data?	All focus groups were audio-recorded using the Zoom recording feature and an external audio tape recorder as backup	Methods
Were field notes made during and/or after the interview or focus group?	Yes, field notes were made during the focus group but were not incorporated as data in analysis	Not reported in manuscript
What was the duration of the interviews or focus group?	Focus groups lasted no more than 2 hours (mean= 88 minutes excluding breaks)	Results
Was data saturation discussed?	Yes<br>We stopped scheduling focus groups when the facilitator, observer, and lead investigator agreed that new insights specific to the study objectives were negligible.	Methods
Were transcripts returned to participants for comment and/or correction?	Yes. A question was included in the demographic questionnaire to offer participants opportunity to review the deidentified transcript of their focus group to ensure all identifiers were removed. 14 of 32 (43.75%) participants wanted to review their focus group transcript.	Methods
**Domain 3: analysis and findings**		
Data analysis		
How many data coders coded the data?	Three researchers coded the data	Methods
Did authors provide a description of the coding tree?	No	N/A
Were themes identified in advance or derived from the data?	A hybrid deductive-inductive approach was applied to coding and theme generation - derived from the focus group guide topics and from the data	Methods
What software, if applicable, was used to manage the data?	NVivo12	Methods
Did participants provide feedback on the findings?	No, participants were not asked to provide feedback on the findings.	Not reported

Reporting		
Were participant quotations presented to illustrate the themes/findings? Was each quotation identified? e.g., participant number	Yes, quotes were identified by participant focus group number and participant number	Results (in-text quotations)
Was there consistency between the data presented and the findings?	Yes	Results
Were major themes clearly presented in the findings?	Yes	Results
Is there a description of diverse cases or discussion of minor themes?	Yes	Results
