# Supplementary material for: “Sepsis brought him to his knees”: exploring the lived experiences and perspectives of sepsis survivors and family members to inform a sepsis public education campaign in Canada
Source: BMC Public Health. 2025 Mar 31;25:1211. doi: 10.1186/s12889-025-22344-9 (PMC11956199; doi:10.1186/s12889-025-22344-9)
Supplement: Supplementary file 3 — Supplementary Material 3 [file 12889_2025_22344_MOESM3_ESM.pdf]

Additional File 2. Semi-Structured Focus Group Guide

**Section 1. Personal Experiences with Sepsis**
1. Who was familiar with sepsis before you got sepsis?<br>a. If yes, can you tell us how you knew about sepsis?
2. If comfortable, can you tell us about how you got sepsis (circumstances that led to sepsis)?
3. At what point did you know, or were told, that you had sepsis?
4. How has sepsis impacted your life?
5. How would you describe your interactions with healthcare providers? This may be first responders such as paramedics, doctors and nurses in the hospital, or in the community.
6. Can you remember how sepsis was described by your care team?<br>a. What did they tell you (what words were used (language))?
7. Did you understand what you were told about your condition?
8. Is there anything in your interactions/communications with healthcare providers that you wish would have happened differently? (e.g., is there anything that you wish you would have been told? What do you wish healthcare providers would say to patients with sepsis to better inform and prepare patients?)
9. Did you get the information that you needed to help understand your condition? (Informational needs versus emotional or physical support needs)
10. How did you use the information you found or received? Were there gaps, and if so, what were the gaps?
11. What healthcare provider made the most impact on your sepsis experience and why?
**Section 2. Perspectives on Raising Awareness**
1. Sepsis Canada is developing a campaign to raise public awareness of sepsis. What do you think are the main messages that we should emphasize about sepsis?<br>a. Is there anything from your own experiences that think could be helpful in our public health campaign about sepsis (and prevention of sepsis)?
2. Do you think there are priority audiences for a public awareness campaign? (are there particular groups of people that we should target for education about sepsis?)
3. Can you think of potential "calls to action" that might be effective campaign slogans – i.e., What action do we want people to take to reduce their chances of developing sepsis?<br>a. What might be barriers in getting people to becoming knowledgeable about sepsis?
4. What would be important channels to use to communicate messages to have a broad reach?<br>a. If you were looking for information about sepsis, what would you do? Or, if you didn't know about sepsis, where do you think you'd likely see information about it?
